# Very High Levels of Violent Discipline Among Children in Afghanistan: Prevalence, Determinants, and Socioeconomic Disparities

**DOI:** 10.21203/rs.3.rs-6228527/v1

**Published:** 2025-03-21

**Authors:** Sayed Ataullah Saeedzai, Jess Ghannam, Ali Mirzazadeh

**Affiliations:** University of California San Francisco

**Keywords:** Violent discipline, Child abuse, Physical punishment, Psychological aggression

## Abstract

**Introduction:**

Violent discipline affects millions of children globally, often justified as essential to child-rearing. This study examines the prevalence and determinants of any physical punishment, severe physical punishment, and psychological aggression towards children among Afghan caregivers.

**Method:**

We analyzed data from the Afghanistan Multiple Indicator Cluster Survey (MICS) 2022–23, which included 23,568 households across all 34 provinces. Information on violent discipline was gathered for children aged 1–14 years. Analyses were adjusted for complex survey design, with Poisson regression assessing predictors of aggression towards children by caregivers, such as language, education, economic status, and child disabilities.

**Results:**

Findings revealed that 81.2% of Afghan children faced physical punishment, with higher rates in Pashto-speaking households (85.1%) and homes with younger heads (82.5%). Severe punishment affected 58.5% of children, especially in rural areas (60.6%) and Pashto-speaking households (65.0%). Psychological aggression impacted 82.8% of children, particularly those with disabilities (89.2%). Maternal education and economic status were protective factors, reducing physical punishment rates from 81.6% among uneducated mothers to 65.0% among educated mothers (p-value < 0.001), and from 81.9% in the poorest to 75.6% in the wealthiest households (p-value < 0.001).

**Conclusion:**

High rates of violent discipline of children in Afghanistan are concerning, especially among those families with low education, high poverty, and child disabilities. Findings highlight an urgent need for targeted interventions to protect children now and avoid lifelong consequences.

## Introduction

Violent discipline remains a pervasive issue worldwide, even among very young children [1]. Approximately 400 million children under the age of five, representing 60% of this age group globally, are regularly subjected to psychological aggression or physical punishment at home [1]. Out of these, around 330 million children experience physical punishment [2]. Harmful social norms that support violent methods of child discipline persist, with over a quarter of mothers and primary caregivers believing that physical punishment is necessary for properly raising and educating children [2].

Child abuse is a pervasive issue in both South and Central Asia, with widespread occurrences of psychological aggression and physical punishment. In the 2019 Multiple Indicator Cluster Survey (MICS) in Pakistan’s Khyber Pakhtunkhwa province, about three-quarters of children experienced psychological aggression, and a similar proportion faced physical punishment [3]. Baluchistan showed somewhat lower rates, with roughly half of children subjected to psychological aggression and physical punishment [4]. Punjab exhibited slightly higher figures in 2017, with over 73% of children facing psychological aggression and over 80% experiencing violent discipline [5]. In Bangladesh’s MICS in 2019, the situation was particularly severe, with over 85% of children enduring psychological aggression and nearly 90% subjected to violent forms of discipline [6]. In Central Asia, particularly in Uzbekistan and Turkmenistan, the figures showed that 25% and 48.8% of children, respectively, endured physical punishment, and more than half were subjected to psychological aggression, though severe physical punishment was reported less frequently [7, 8].

Understanding the determinants of harmful parenting behaviors is needed to foster healthier developmental environments for children. Children with functional difficulties related to mental health or cognitive impairments are at a significantly increased prevalence of experiencing violent parental discipline [9]. This exposure correlates with various negative outcomes, including poor mental and physical health, impaired cognitive and socio-emotional development, and academic challenges. Both boys and girls across different economic status face similar prevalence of physical punishment [2]. The evidence from many countries highlights that violent discipline is prevalent even among the youngest children [1]. A systematic review, mainly from high-income countries, found that 94% of studies reported significant links between maternal spanking or corporal punishment and negative child behavior and development, either immediately or later. These practices also played a role as mediators or moderators in models explaining early childhood behavioral issues [10]. The physical punishment and psychological aggressions are associated with learning difficulties, anxiety, and depression in the study also identified in Pakistan [11].

This paper aims to identify the prevalence of various violent child-rearing behaviors among caregivers in Afghanistan and explore the determinants influencing these practices. While several MICS surveys have been conducted in Afghanistan over the years, there has been limited further investigation into violent child-rearing methods, and the existing literature remains sparse. Understanding the socio-demographic and cultural factors that shape violent child-rearing behaviors is critical, as it can inform strategies for promoting positive parenting practices and mitigating harmful disciplinary methods. Such research is particularly significant in the Afghan context, where cultural norms and socio-economic conditions can profoundly impact child development and family dynamics. Addressing this gap is essential for advancing knowledge and fostering healthier parenting practices in Afghanistan. The objective of this study is to identify the prevalence and determinants of child physical punishment and psychological aggression practices at home. Specifically, it aims to explore the socio-economic and contextual factors associated with child physical punishment, severe physical punishment, psychological aggression, and any form of physical or psychological aggression.

### Ethical considerations

The MICS protocol was reviewed and approved by the NSIA and UNICEF, ensuring compliance with ethical guidelines. Informed consent was obtained from all participants, to help ensure voluntary participation and understanding of the study’s aims and procedures. Confidentiality of participant data was maintained.

## Methods

### Study design

This paper used a secondary analysis of the Afghanistan MICS 2022/23 survey data. The focus is on identifying the determinants of physical and psychological violent discipline of children at home. The MICS 2022/23 study was conducted in all 34 provinces of Afghanistan by the United Nations Children’s Fund (UNICEF) in collaboration with the National Statistics and Information Authority (NSIA). The survey utilized a two-stage cluster sampling approach, with a sample size of 23,568 households across 982 enumeration areas in 34 provinces. Security concerns led to the exclusion of 10 selected enumeration areas.

### Study outcomes

Child violence information was obtained from children aged 1 to 14 years in the household. For households with multiple children in this age range, one child was randomly selected from 5 to 14 years, all children 1 to 5, and their caregivers were interviewed. Physical violence included various forms of physical harm inflicted on children by caregivers, such as shaking, spanking, hitting or slapping on the bottom with a bare hand, hitting with a belt, hairbrush, stick, or other hard objects, hitting or slapping on the face, head, or ears, hitting or slapping on the hand, arm, or leg, and beating the child repeatedly with maximum force. Severe physical punishment specifically referred to hitting or slapping on the face, head, or ears, and beating the child as hard as possible multiple times. Psychological aggression encompassed behaviors like shouting, yelling, screaming, or calling the child derogatory names such as “dumb” or “lazy.” The category of “any physical punishment or psychological aggression” included children who have experienced at least one form of physical punishment or psychological aggression.

### Study predictors

The independent variables in this study included the language spoken by the head of household, mother’s education, household head’s education, and household size (categorized as members less than or equal to 5,6 to 8,9 to 12, and above 12). Economic status was divided into wealth quintiles based on household assets, while area was categorized as urban or rural. Other variables included the household head’s age, mother’s belief that physical punishment is necessary, child’s sex, child’s age, and functional difficulties. Functional difficulties covered multiple domains. For children under five, these included seeing, hearing, walking, fine motor skills, communication, learning, playing, and behavior control. For children aged 5–17 years, domains included seeing, hearing, walking, self-care, communication, learning, remembering, concentrating, accepting change, controlling behavior, making friends, anxiety, and depression [12].

## Statistical analysis

Data management involved constructing relevant outcome and independent variables as defined above as reported in the MICS [12]. The data was obtained from children under five, children aged 5 to 17 years, and household datasets. Analysis was conducted using STATA version 18, with adjustments made for the complex survey design, including stratification by province and area, and clustering by enumeration areas, and appropriate weighting applied to adjust for over sampling. Descriptive statistics (i.e., numbers, proportions in sample and population) summarized key variables. The distribution of all four outcome variables of physical punishment, severe physical punishment, psychological aggression, and any physical or psychological aggression distribution were assessed by independent variables and p-values were reported accordingly. Poisson regression models were used to assess the association between independent variables and the occurrence of physical punishment, severe physical punishment, psychological aggression, and any physical or psychological aggression, adjusting for survey design effects. A stepwise approach was applied to identify significant predictors of each outcome. A univariate analysis was first conducted to identify predictors with p-value < 0.2 to be included as potential predictors in the multivariable Poisson regression model. A stepwise approach was used to retain only those predictors with a p-value ≤ 0.05 in the final model.

## Results

### Household characteristics

Table 1 presents a snapshot of households and children who participated in the 2023 MICS in Afghanistan, offering insights into the demographic, educational, and socio-economic landscape based on a sample of 42,558 individuals in the sampled households.

Most household have children 1 to 14 years old. The head of households in Afghanistan predominantly speak Pashto (48.0% of the sample), followed by Dari (40.1%), with smaller percentages speaking Uzbeki (7.3%), Turkmani (1.6%), Nooristani (0.5%), Pahshaie (1.2%), Balochi (0.4%), and other minority languages (0.3%). Household heads are typically in the working-age range, with the largest group being between 25 and 39 years old (37.4.0%). A nearly as large proportion were aged 40 to 54 years (37.0%), while those over 70 represent were 6.8%. Afghanistan remains largely rural, with 76.8% of the sampled households located in rural areas and only 23.2% living in urban settings.

Education levels among household heads were low, with 65.8% having no formal education or only basic pre-primary schooling. Among mothers, 85.2% lacked formal education. Only a small percentage of both household heads and mothers had attained higher education degrees. Most households are que large, with typically 6 to 8 members (33.4%) or 9 to 12 members (37.7%) and some with over 12 members (22.5%).

### Children characteristics

The sample and population had boys making up 51.3% of the children and girls representing 48.7%. Among children of 1 to 14 years, those aged 1 and 2 years old were 14.8%, 3 to 4 years were 16.4%,5 to 9 make up 37.7%, and 10 to 14 years were 31.2%. Functional difficulties among children were reported in 31.5% of the sample.

### Any physical punishment

The data in Table 2 indicates that physical punishment affected 81.2% of children aged 1 to 14 at least once in the month prior to the survey. This prevalence varies significantly across linguistic groups, with Pashto-speaking households reporting the highest rate at 85.1% and Turkmani-speaking households the lowest at 58.6% (p < 0.05). When adjusted for other factors (Table 3), children in Pashto-speaking households have a slightly increased prevalence (adjusted prevalence ratio [PR] 1.07) compared to those in Dari-speaking households, while Turkmani-speaking households demonstrate a notably lower likelihood (adjusted [PR] 0.74).

Household characteristics further influence the prevalence of physical punishment. Younger heads of households, specifically those aged 25 to 39 years, report a prevalence of 82.5%, slightly higher than that in households headed by individuals aged 70+ (80.5%) (Table 2). Maternal education shows a protective effect, with physical punishment prevalence at 81.6% among children of mothers with no formal education, 77.4% for those with secondary education, and 65% for mothers with higher education (p < 0.001). Adjusted analyses reflect these findings, with the lowest adjusted PR (0.89) among children of highly educated mothers and a higher PR (1.06) for those with primary education (Table 3).

Economic status plays a similarly protective role, with physical punishment rates decreasing from 81.9% among children in the poorest quintile to 75.6% in the wealthiest (p < 0.001). This association remains significant in the adjusted, with children from the richest households having an adjusted PR of 0.94. Maternal attitudes also strongly influence outcomes; in households where mothers believe physical punishment is necessary, 92.7% of children experience it, compared to 76.4% in households where mothers oppose it.

Children with functional difficulties face an increased prevalence of physical punishment at 88.4%, compared to 81.1% among children without such difficulties (p < 0.001). Age is another predictive factor; children aged 3 to 4 and 5 to 9 years report the highest prevalence of physical punishment at 85.2% and 87.5%, respectively, with adjusted PRs of 1.25, 1.29, and 1.14 for children aged 3–4,5–9, and 10–14 years compared to those aged 1–2 years. Gender differences are smaller, with male children experiencing physical punishment at a prevalence of 82.1% versus 80.1% for females (p < 0.00

### Severe physical punishment

Severe physical punishment affects 58.5% of Afghan children aged 1 to 14 years. Pashto-speaking households have the highest prevalence at 65.0%, while Nooristani and Turkmani-speaking households have the lowest rates at 27.1% and 31.3%, respectively (p < 0.001). Adjusted analyses further indicate that Pashto-speaking households have an increased prevalence of severe punishment (adjusted PR 1.20) relative to Dari-speaking (reference group) households, while children in Turkmani-speaking households show a significantly lower likelihood (adj PR 0.57) and Nooristani-speaking households even lower (adj PR 0.48).

Rural households report higher rates of severe physical punishment at 60.6% compared to 51.5% in urban areas (p < 0.001) and an adjusted PR of 1.18. The child’s age also significantly impacts the likelihood of severe physical punishment, with children aged 5 to 9 years showing the highest prevalence at 65.5%. Relative to children aged 1 to 2 years old (reference group), those aged 3 to 4, 5 to 9, and 10 to 14 years have increased likelihoods of experiencing severe punishment (adjusted PRs 1.44, 1.55, and 1.33, respectively).

Children of mothers with higher education face a substantially lower prevalence of severe physical punishment at 34.3% (p < 0.001, adjusted PR 0.75). Mothers with primary or no formal education report significantly higher prevalence of severe punishment of their children at 81.6% and 82.9%, respectively. This protective trend also applies to the education level of the household head, where those with secondary or higher education show reduced PRs (0.89 and 0.87, respectively) for severe physical punishment of their children.

Children with functional difficulties are at increased prevalence for severe physical punishment at 67.6% compared to 58.4% among those without such difficulties (p < 0.001). Households with more than 12 members exhibit a higher prevalence of severe physical punishment (adj PR 1.14) compared to households with five or fewer members.

Furthermore, severe physical punishment prevalence is higher in households where mothers consider physical punishment necessary, at 75.0% compared to 53.6% among mothers who oppose it. Further, children in the wealthiest quintile of households show reduced prevalence of severe physical punishment (adj PR 0.86) compared to the poorest (reference).

### Psychological aggression

Psychological aggression was reported in 82.8% of Afghan households. Pashto-speaking households show the highest prevalence at 85.4% (adjusted PR 1.04), compared to Dari-speaking households at 81.1%, and Turkmani-speaking households at 66.2% (p < 0.001, adjusted PR 0.80). Notably, Nuristani-speaking households show the highest adjusted PR of 1.08 for psychological aggression.

Psychological aggression is more common in rural households (83.7%) compared to urban ones (79.8%) (p < 0.001), with rural children facing higher adjusted prevalence (adjusted PR 1.04). Children whose mothers have secondary or higher education experience reduced psychological aggression, with adjusted PRs of 0.92 and 0.90, respectively. Children of mothers with no formal education experience a higher prevalence of psychological aggression at 83.4%; with higher education among mothers the prevalence is 70.0%.

The children in the poorest quintile households face higher levels of psychological aggression (82.4%) compared to those in the wealthiest quintile (78.7%) (p < 0.001, adjusted PR 0.97). Age also influences the likelihood of psychological aggression, with children aged 3 to 4, 5 to 9, and 10 to 14 years showing higher prevalence (adjusted PRs of 1.28, 1.34, and 1.26, respectively), relative to those aged 1 to 2 years.

About 89.2% of children with difficulties facing psychological aggression, compared to 84.2% of those without (p < 0.001). Children without functional difficulties are less likely to face psychological aggression, reflected in a crude PR of 0.94. Lastly, households where mothers oppose physical punishment show a greater protective effect against psychological aggression (crude PR = 0.86).

### Any physical or psychological aggression

The prevalence of any form of violent discipline, physical or psychological, affects 87.6% of Afghan children. Pashto-speaking households report the highest prevalence at 90.3%, while Turkmani-speaking households show a notably lower prevalence at 71.1% (p < 0.001, adjusted PR 1.04 and 0.82, respectively, compared to Dari-speaking). Children of mothers who have secondary or higher education experiencing less aggression (84.0% and 77.4%, respectively) than those whose mothers has no or lack formal education (87.8%, p < 0.001). In adjusted analyses, households with the most educated mothers demonstrate lower aggression (adjusted PR 0.92). Child’s age also impacts exposure to aggression; children aged 3 to 4, 5 to 9, and 10 to 14 years have adjusted PRs of 1.21, 1.24, and 1.18, respectively, compared to those aged 1 to 2 years.

## Discussion

The study highlights the troubling extent of child punishment and aggression within Afghan households, with 81.2% of children aged 1 to 14 years experiencing physical punishment, 58.5% subjected to severe forms, and 82.8% enduring psychological aggression. This places Afghanistan among the highest in the region for violent discipline practices especially severe physical punishment, with physical and psychological aggression rates significantly higher than those of neighboring countries. In comparison, rates in Pakistan vary, with around 73–77% of children in Khyber Pakhtunkhwa and Punjab exposed to psychological aggression and over 70% facing physical punishment [3, 5], while Baluchistan shows notably lower figures, with approximately half of children experiencing these forms of discipline [4]. In Bangladesh, although the levels of psychological aggression are similarly high at 85%, violent discipline affects nearly 89% of children [6]. In Central Asia, particularly in Uzbekistan and Turkmenistan, between 25% and 49% of children endure physical punishment, and more than half are subjected to psychological aggression, though severe punishment is less common (1 to 4%) compared to finding of this study. These comparisons underscore Afghanistan’s alarmingly high rates of violent discipline, necessitating urgent attention alongside regional efforts to address harmful child-rearing practices [7, 8].

An important findings is head of household language as a proxy for ethnicity effect on violent discipline in Afghanistan, as indicated by the household head’s language, plays a critical role in predicting levels of punishment. Pashto-speaking households show the highest rates of physical, severe physical. In contrast, Turkmen- and Uzbek-speaking households report the lowest levels of severe physical punishment. This finding is consistent with the lower rates of child punishment in Uzbekistan and Turkmenistan, where these ethnic groups live near border areas [7, 8]. Smaller ethnic groups such as the Pashaie, Nuristani, and Balochi exhibit high levels of punishment, although Nuristanis report the least severe physical punishment.

Education of the mother and the household head is another significant factor influencing child punishment. The highest level of education was linked to lower rates of physical and psychological aggression. A similar finding was noted in a study of children in Malatya, East Anatolia, in that mothers with higher education was associated with nonviolent child discipline approaches [13].

We also found that children with functional difficulties are at a higher prevalence of experiencing punishment. These children faced greater levels of physical punishment, severe physical punishment, and psychological aggression, making them more vulnerable to all forms of maltreatment, plus having already functionality problems. These results show awareness issues among caregivers to the ones having functional difficulties. Similar results found in UNICEF’s Multiple Indicator Cluster Surveys for 206, 147 children aged 2–14 across 17 countries study, revealing that children with disabilities are significantly more likely to experience various forms of violent parental discipline compared to their non-disabled peers [9]. Addressing these factors through culturally sensitive interventions is essential to reducing child punishment and improving child welfare. A study from Pakistan found that severe physical, psychological, and emotional disciplinary methods were associated with increased challenges in learning, memory retention, behavior control, anxiety, depression, and social interactions[11], as such behaviors will further exacerbate the functional difficulties.

Age and gender also play crucial roles in determining the level of punishment. Children aged 1 to 2 years are not spared from physical and severe punishment, but aggression peaks among children aged 5 to 9 years, followed by those aged 3 to 4 years, other studies also showed that younger children also faced similar violence [1]. Both boys and girls are subjected to physical punishment, though boys experience higher punishment than girls in Afghanistan. A study conducted in eight Asia-Pacific countries reported that girls in the study had a higher odds (OR 1.22), contrasting with our findings [14].

Maternal beliefs, poverty, rural residence, and family size are critical factors influencing physical and psychological aggression in Afghanistan. Poverty and rural living conditions often limit access to education and child-rearing resources, which can lead to the normalization of physical punishment and psychological aggression as a disciplinary measure, similar results reported in high nonviolent discipline behavior in rural Bangladesh, the life time psychological abuse of 97% [15]. Larger families may face additional pressures, with limited attention and resources contributing to harsher parenting practices, A study conducted in eight Asia-Pacific countries showed similar results, with larger family sizes (7 or more members) associated with an increased odds of violent discipline (OR 1.43) [14]. Furthermore, when mothers believe that physical punishment is a necessary part of raising children, it reinforces and perpetuates aggressive behavior, the results are consistent in other studies conducted in Egypt (OR 3.3) for severe physical punishment [16].

This study has notable strengths, including a large sample size that enabled robust analysis, as well as national and provincial representation that provided insights into the burden of physical and psychological aggression against children across Afghanistan. The use of multiple questions to assess different forms of child punishment allowed for a comprehensive exploration of the issue. However, the study has some limitations, particularly the potential for reporting bias, as respondents may underreport violence, especially when it is self-reported by those interviewed and responsible for the punishment. Social desirability bias could further skew responses especially among educated and people living in urban areas.

This study underscores the urgent need for targeted interventions to reduce child punishment in Afghanistan. Ethnicity, education, disability status, and mother believe violent discipline are needed, suggesting that culturally sensitive programs focusing on vulnerable groups, especially those with lower education levels and specific ethnicities, are essential to addressing this issue. To combat the high levels of child punishment in Afghanistan, policy and regulatory development is essential to promote non-violent disciplinary practices. National legislation should explicitly prohibit physical and psychological punishment, with strict enforcement to ensure compliance. Training programs both in person and online must be developed for parents, caregivers, and adults planning to have children, educating them on positive, non-violent child-rearing techniques. Additionally, population-wide awareness campaigns are needed to shift societal norms, emphasizing the harm caused by physical and psychological aggression. Special policies should be crafted to protect children with functional difficulties, who are particularly vulnerable to punishment, ensuring they receive appropriate care and protection. Integrating these elements into Afghanistan’s social setting can create a safer environment for children and reduce violence in child-rearing practices.

## Conclusion

This study reveals an alarming prevalence of child punishment and aggression in Afghanistan, where physical and psychological punishment rates are among the highest in the region. Ethnicity, education, poverty, and maternal beliefs are key determinants of violent child discipline in Afghanistan. Children with functional difficulties are particularly at risk. Addressing child punishment in Afghanistan requires a comprehensive approach, involving legal reforms, awareness campaigns, education, and targeted support for vulnerable children, to promote non-violence and improve overall child welfare.

## Figures and Tables

**Table 1:** Characteristics of households and children enrolled in the Multiple Indicators Cluster Survey (MICS) 2023 (N = 42,558), Afghanistan

Characteristics of households, mothers	n	% (Sample)	% (Population)*
**Head of household Language**			
Dari	15,773	37.0	40.1
Pashto	20,270	47.7	48.6
Uzbeki	3,062	7.2	7.3
Turkmani	730	1.7	1.6
Nooristani	1,130	2.7	0.5
Balochi	343	0.8	0.4
Pashaie	1,030	2.4	1.2
Other Languages	220	0.5	0.3
**Household Head Age**			
11–24 (Youth)	1,925	4.5	3.7
25–39 (Young Adults)	17,877	42	37.4
40–54 (Middle-aged Adults)	13,294	31.2	37.0
55–69 (Older Adults)	6,460	15.2	15.0
70+ (Seniors)	3,002	7.1	6.8
**Living area**			
Urban	6,661	15.6	23.2
Rural	35,965	84.4	76.8
**Education of household head**			
Pre-primary/Early childhood education or none	28,469	66.8	65.8
Primary	4,459	10.5	11.9
Lower Secondary	2,538	6.0	6.7
Upper Secondary	4,091	9.6	8.9
Higher	3,001	7.0	6.6
Don’t know/Missing	68	0.2	0.2
**Mother’s Education**			
Pre-primary/Early childhood education or none	36,193	85.0	85.2
Primary	2,864	6.7	7.2
Lower Secondary	1,201	2.8	2.8
Upper Secondary	1,421	3.3	3
Higher	879	2.1	1.8
**Number of household members**			
≤ 5	5,596	13.2	9.4
6 to 8	14,214	33.4	33.4
9 to 12	13,343	31.4	37.7
> 12	9,405	22.1	22.5
Wealth index quintile			
Poorest	10,522	24.7	22.5
Second	10,162	23.9	21.4
Middle	9,325	21.9	20.2
Fourth	7,478	17.6	19.3
Richest	5,071	11.9	16.6
**Mother/caregiver believes on physical punishment is necessary for proper child-rearing**			
Yes	4,801	11.3	16.3
No	13,623	32.0	39.3
Don’t Know/no opinion	167	0.4	0.1
No Response	8	0.0	0
Missing	23,959	56.3	43.7
**Characteristics of children**			
**Child sex**			
Male	21,979	51.6	51.3
Female	20,647	48.4	48.7
**Child age**			
1–2 years	12,486	29.3	14.8
3–4 years	14,026	32.9	16.4
5–9 years	9,490	22.3	37.7
10–14 years	6,624	15.5	31.2
**Child Functional difficulty**			
Has functional difficulty	7,976	26.5	31.5
Has no functional difficulty	22,164	73.5	68.5

%* Weighted percentage

**Table 2:** Prevalence of physical punishment and psychological aggression at home among children 1–14 years old in Afghanistan, 2023 (N = 42,458)

	Physical punishment					Psychological aggression					Physical punishment or psychological aggression	
	Any			Severe			Any				Any	
	n	(%)	P-value	n	(%)	P-value	n	(%)	P-value	n	(%)	P-value
**Total**	33,184	81.2		22,612	58.5		33,154	82.8		35,771	87.6	
**Characteristics of households, mothers**												
**Head of household language**												
Dari	11,684	78.7	<0.001	7,674	54	<0.001	11,959	81.1	<0.001	12,888	86.2	<0.001
Pashto	16,808	85.1		12,379	65		16,317	85.4		17,707	90.3	
Uzbeki	2,101	72.7		1,264	47.6		2,230	77.6		2,350	80.9	
Turkmani	406	58.6		215	31.3		433	66.2		478	71.1	
Nooristani	931	85.3		285	27.1		953	88.3		1,017	92.2	
Balochi	255	79.5		189	62.1		257	86.4		278	89.2	
Pashaie	848	86.4		517	60.5		853	85.3		892	88.1	
Other Languages	151	70.4		89	45		152	73.9		161	76.8	
**Household head age (years)**												
11–24 (Youth)	1,925	79.9	0.013	945	54.66	0.034	1,480	80.32	0.321	1,599	85.2	0.201
25–39 (Young adults)	17,877	82.5		9,632	60.25		13,924	83.23		15,058	88.0	
40–54 (Middle-aged adults)	13,294	80.4		7,074	57.86		10,410	82.55		11,221	87.2	
55–69 (Older adults)	6,460	80.0		3,425	56.7		5,029	82.32		5,419	87.6	
70+ (Seniors)	3,002	80.5		1,536	57.82		2,311	84.02		2,474	88.2	
**Area**												
Urban	4,930	78.6	0.002	3,251	51.5	<0.001	4923	79.8	<0.001	5380	86.5	0.018
Rural	15,898	81.9		19,361	60.6		28231	83.7		30391	88.0	
**Mother’s education**												
Pre-primary early childhood education or none	28,461	81.6	<0.001	19,594	59.7	<0.001	28,394	83.4	<0.001	30,596	87.8	<0.001
Primary	2,285	82.9		1,608	60		2,306	84.7		2,453	89.4	
Lower secondary	891	78.5		568	51.5		886	78.4		971	85.8	
Upper secondary	1,010	77.4		556	39.7		998	72.6		1,116	84.0	
Higher	537	65.0		286	34.3		570	70.0		635	77.4	
**Wealth index quintile**												
Poorest	8,288	81.9	<0.001	5,772	61.5	<0.001	8,112	82.4	<0.001	8,850	87.5	0.002
Second	8,052	83.4		5,539	62.7		7,987	84.2		8,646	88.7	
Middle	7,362	82.4		5,021	60.9		7,361	84		7,901	88	
Fourth	5,789	81.3		3,868	55.8		5,884	83.9		6,267	88.2	
Richest	3,693	75.6		2,412	49.2		3,810	78.7		4,107	84.9	
**Mother/caregiver believes on physical punishment is necessary for proper child-rearing**												
Yes	4377	92.7	<0.001	3,466	75.0	<0.001	4368	93.2	<0.001	4554	96.1	<0.001
No	10112	76.4		6,604	53.6		19365	80.0		11214	85.1	
**Education of household head**												
Pre-primary/Early childhood education or none	22,404	82.4	<0.001	15,466	60.87	<0.001	22,275	83.6	<0.001	24,022	88.1	0.037
Primary	3,495	79.5		2,509	57.58		3,561	83.6		3,796	87.4	
Lower secondary	1,991	81.0		1,340	56.34		2,007	82.3		2,154	87.9	
Upper secondary	3,080	77.0		1,968	51.01		3,091	79.9		3,359	85.1	
Higher	2,214	77.6		1,329	48.09		2,220	78.0		2,440	86.0	
**Number of household members**												
≤ 5	4,206	77.61	<0.001	2,805	52.45	<0.001	4,254	79.23	0.008	4,595	84.43	0.003
6 to 8	11,042	80.17		7,475	56.61		11,088	82.77		11,982	87.6	
9 to 12	10,392	81.23		7,100	59.04		10,381	83.07		11,225	87.74	
> 12	7,544	83.93		5,232	62.83		7,431	83.86		7,969	88.59	
**Characteristics of children**												
**Sex**												
Male	17286	82.1	0.002	11,968	60.3		17189	83.5	0.01	18548	88.0	0.117
Female	15898	80.1		10,644	56.5		15965	82.1		17223	87.2	
**Age (years)**												
1–2	8,243	67.9	<0.001	4,946	42.3	<0.001	7,997	34.3	<0.001	9,040	74.2	<0.001
3–4	11,801	85.2		8,213	60.9		11,578	16.1		12,423	89.5	
5–9	8,150	87.5		5,948	65.5		8,206	11.6		8,631	92	
10–14	4,990	77.7		3,505	56.4		5,373	16.6		5,677	87.6	
**Child Functional difficulty**												
Has functional difficulty	6813	88.4	<0.001	5071	67.6	<0.001	6822	89.2	<0.001	7189	92.5	<0.001
Has no functional difficulty	18128	81.1		12595	58.4		18335	84.2		19542	88.7	

Weighted % and P-value, the numbers are unweighted

**Table 3:** Prevalence ratio to assess determinants of physical punishment and psychological aggression at home among children 1–14 years in Afghanistan, 2023 (N = 42,558)

	Physical punishment				Any psychological aggression		Any physical punishment or psychological aggression	
	Any		Severe					
	Crude PR	Adj. PR			Crude PR	Adj. PR	Crude PR	Adj. PR
	Est. (95CI%)	Est. (95CI%)	Est. (95CI%)	Est. (95CI%)	Est. (95CI%)	Est. (95CI%)	Est. (95CI%)	Est. (95CI%)
**Head of household language**								
Dari	1	1	1	1	1	1	1	1
Pashto	1.08 (1.06, 1.11)	1.07 (1.05, 1.10)	1.20 (1.15, 1.26)	1.15 (1.09, 1.20)	1.05 (1.03, 1.07	1.04 (1.02, 1.06)	1.05 (1.03, 1.06	1.04 (1.03, 1.06)
Uzbeki	0.92 (0.88, 0.97)	0.92 (0.88, 0.96)	0.88 (0.81, 0.96)	0.86 (0.79, 0.93)	0.96 (0.92, 1.00)	0.95 (0.91, 0.99)	0.94 (0.91, 0.97)	0.94 (0.90, 0.97)
Turkmani	0.75 (0.65, 0.86)	0.74 (0.64, 0.85)	0.58 (0.46, 0.74)	0.57 (0.44, 0.72)	0.82 (0.73, 0.91)	0.80 (0.72, 0.90)	0.82 (0.74, 0.92)	0.82 (0.74, 0.92)
Nooristani	1.08 (1.02, 1.14)	1.06 (1.01, 1.12)	0.50 (0.40, 0.74)	0.48 (0.39, 0.59)	1.09 (1.04, 1.14)	1.08 (1.03, 1.13)	1.07 (1.03, 1.11)	1.06 (1.03, 1.10)
Balochi	1.01 (0.87, 1.14)	1.01 (0.89, 1.15)	1.15 (0.98, 1.27)	1.09 (0.91, 1.31)	1.07 (1.01, 1.12)	1.06 (1.00, 1.12)	1.03 (0.99, 1.08)	1.04 (0.99, 1.08)
Pashaie	1.10 (1.05, 1.15)	1.08 (1.03, 1.13)	1.12 (0.99, 1.14)	1.06 (0.93, 1.21)	1.05 (1.01, 1.10)	1.04 (1.00, 1.09)	1.02 (0.98, 1.06)	1.02 (0.98, 1.06)
Other Languages	0.89 (0.72, 1.11)	0.89 (0.73, 1.09)	0.83 (0.61, 1.14)	0.82 (0.62, 1.08)	0.91 (0.74, 1.12)	0.90 (0.74, 1.09)	0.89 (0.74, 1.07)	0.89 (0.74, 1.06)
**Household head age (years)**								
11–24 (Youth)	1.00		1	1	1		1	
25–39 (Young adults)	1.03 (0.99, 107)		1.10 (1.03, 1.18)	1.08 (1.00, 1.16)	1.04 (0.99, 1.08)		1.03 (1.00, 1.06)	
40–54 (Middle-aged adults)	1.01 (0.97, 1.04)		1.06 (0.98, 1.14)	1.00 (0.93, 1.08)	1.03 (0.98, 1.07)		1.02 (0.99, 1.06)	
55–69 (Older adults)	1.00 (0.96, 1.04)		1.04 (0.96, 1.12)	0.99 (0.91, 1.07)	1.02 (0.98, 1.07)		1.03 (0.99, 1.06)	
70+ (Seniors)	1.01 (0.96, 1.05)		1.06 (0.96, 1.17)	0.98 (0.89, 1.09)	1.05 (1.00, 2.09)		1.04 (1.00, 1.07)	
**Area**								
Urban	1		1		1	1	1	1
Rural	1.04 (1.01, 1.07)		1.18 (1.10, 1.25)		1.05 (1.02, 1.08)	1.04 (1.00, 1.07)	1.02 (1.00, 1.04)	1.02 (1.00, 1.04)
**Mother’s Education**								
Pre-primary/Early childhood education or none	1	1	1	1	1	1	1	1
Primary	1.02 (0.99, 1.05)	1.06 (1.03, 1.09)	1.00 (0.94, 1.07)	1.12 (1.05, 1.20)	1.01 (0.99, 1.04)	1.05 (1.03, 1.08)	1.02 (1.00, 1.04)	1.04 (1.02, 1.07)
Lower secondary	0.96 (0.91, 1.01)	1.02 (0.96, 1.07)	0.86 (0.77, 0.96)	1.00 (0.89, 1.12)	0.94 (0.89, 0.99)	0.98 (0.93, 1.04)	0.98 (0.94, (1.02)	1.01 (0.97, 1.05)
Upper secondary	0.95 (0.90, 1.0)	1.01 (0.96, 1.07)	0.67 (0.59, 0.75)	0.82 (0.72, 0.93)	0.87 (0.81, 0.93)	0.92 (0.86, 0.99)	0.96 (0.92, 0.99)	1.00 (0.96, 1.03)
Higher	0.80 (0.72, 0.88)	0.89 (0.78, 0.95)	0.57 (0.48, 0.69)	0.75 (0.63, 0.90)	0.83 (0.78, 0.90)	0.90 (0.84, 0.97)	0.88 (0.83, 0.94)	0.92 (0.87, 0.99)
**Wealth index quintile**								
Poorest	1	1	1	1	1	1	1	
Second	1.02 (1.0, 1.04)	1.02 (1.00, 1.04)	1.02 (0.98, 1.06)	1.03 (0.98, 1.20)	1.02 (1.00, 1.04)	1.03 (1.00, 1.05)	1.01 (1.00, 1.03)	
Middle	1.01 (0.98, 1.03)	1.02 (0.99, 1.04)	0.99 (0.95, 1.04)	1.00 (0.95, 1.05)	1.02 (1.00, 1.04)	1.03 (1.00, 1.05)	1.00 (0.99, 1.03)	
Fourth	0.99 (0.97, 1.02)	1.00 (0.97, 1.03)	0.91 (0.86, 0.96)	0.93 (0.88, 0.98)	1.02 (0.99, 1.05)	1.04 (1.01, 1.06)	1.00 (0.99, 1.03)	
Richest	0.92 (0.89, 0.95)	0.94 (0.91, 0.97)	0.80 (0.75, 0.86)	0.86 (0.81, 0.93)	0.96 (0.92, 0.99)	1.00 (0.98, 1.04)	0.97 (0.95, 0.99)	
**Mother/caregiver believes on physical punishment is necessary for proper child-rearing**								
Yes	1		1		1		1	
No	0.82 (0.81, 0.84)		0.72 (0.69, 0.75)		0.86 (0.84, 0.87)		0.88 (0.87, 0.90)	
**Education of household head**								
Pre-primary/Early childhood education or none	1		1	1	1		1	
Primary	0.97 (0.94, 1.00)		0.95 (0.89, 1.00)	0.97 (0.92, 1.03)	1.00 (0.97, 1.03)		0.99 (0.97, 1.02)	
Lower secondary	0.98 (0.95, 1.02)		0.93 (0.86, 1.00)	0.95 (0.89, 1.03)	0.99 (0.95, 1.02)		1.00 (0.97, 1.02)	
Upper secondary	0.94 (0.90, 0.97)		0.84 (0.79, 0.89)	0.89 (0.84, 0.95)	0.96 (0.93, 0.98)		0.97 (0.94, 0.99)	
Higher	0.94 (0.91, 0.98)		0.80 (0.73, 0.85)	0.87 (0.81, 0.93)	0.93 (0.89, 0.98)		0.98 (0.95, 1.00)	
**Number of household members**								
≤ 5	1		1	1	1		1	
6 to 8	1.03 (1.01, 1.06)		1.08 (1.03, 1.13)	1.02 (0.97, 1.07)	1.04 (1.02, 1.7)		1.04 (1.02, 1.06)	
9 to 12	1.05 (1.02, 1.08)		1.13 (1.7, 1.18)	1.06 (1.00, 1.11)	1.05 (1.02, 1.08)		1.04 (1.02, 1.06)	
> 12	1.08 (1.05, 1.11)		1.20 (1.13, 1.27)	1.14 (1.07, 1.21)	1.06 (1.03, 1.09)		1.05 (1.02, 1.07)	
**Sex**								
Male	1	1	1	1	1		1	
Female	0.98 (0.96, 0.99)	0.98 (0.96, 0.99)	0.94 (0.91, 0.97)	0.94 (0.91, 0.97)	0.98 (0.97, 1.00)		0.99 (0.98, 1.00)	
**Age (years)**								
1–2	1	1	1	1	1	1	1	1
3–4	1.25 (1.23, 1.28)	1.25 (1.23, 1.28)	1.44 (1.40, 1.49)	1.43 (1.39, 1.48)	1.28 (1.25, 1.30)	1.28 (1.25, 1.30)	1.20 (1.19, 1.23)	1.21 (1.18, 1.23)
5–9	1.29 (1.26, 1.32)	1.29 (1.26, 1.31)	1.55 (1.49, 1.61)	1.54 (1.48, 1.60)	1.35 (1.32, 1.38)	1.34 (1.31, 1.37)	1.24 (1.22, 1.26)	1.24 (1.22, 1.26)
10–14	1.14 (1.12, 1.17)	1.14 (1.11, 1.17)	1.33 (1.28, 1.39)	1.32 (1.27, 1.38)	1.27 (1.24, 1.30)	1.26 (1.23, 1.30)	1.18 (1.16, 1.20)	1.18 (1.16, 1.20)
**Child functional difficulty**								
Has functional difficulty	1		1		1		1	
Has no functional difficulty	0.92 (0.90, 0.93)		0.86 (0.83, 0.89)		0.94 (0.93, 0.96)		0.96 (0.95, 0.97)	

## Data Availability

the data for the Afghanistan MICS 2022/2023 study is publicly available on the UNICEF MICS website at https://mics.unicef.org/
